# Pedestrian Dead Reckoning-Assisted Visual Inertial Odometry Integrity Monitoring

**DOI:** 10.3390/s19245577

**Published:** 2019-12-17

**Authors:** Yuqin Wang, Ao Peng, Zhichao Lin, Lingxiang Zheng, Huiru Zheng

**Affiliations:** 1 School of Informatics, Xiamen University, Xiamen 361005, China; 23320171153187@stu.xmu.edu.cn (Y.W.); 23320181154318@stu.xmu.edu.cn (Z.L.); lxzheng@xmu.edu.cn (L.Z.); 2 School of Computing, Ulster University, Newtownabbey BT37 0QB, UK; h.zheng@ulster.ac.uk

**Keywords:** visual-inertial odometer, pedestrian dead reckoning, autonomous integrity monitoring

## Abstract

Visual inertial odometers (VIOs) have received increasing attention in the area of indoor positioning due to the universality and convenience of the camera. However, the visual observation of VIO is more susceptible to the environment, and the error of observation affects the final positioning accuracy. To address this issue, we analyzed the causes of visual observation error that occur under different scenarios and their impact on positioning accuracy. We propose a new method of using the short-time reliability of pedestrian dead reckoning (PDR) to aid in visual integrity monitoring and to reduce positioning error. The proposed method selects optimized positioning by automatically switching between outputs from VIO and PDR. Experiments were carried out to test and evaluate the proposed PDR-assisted visual integrity monitoring. The sensor suite of experiments consisted of a stereo camera and an inertial measurement unit (IMU). Results were analyzed in detailed and indicated that the proposed system performs better for indoor positioning within an environment that contains low illumination, little background texture information, or few moving objects.

## 1. Introduction

In the modern world, people are becoming increasingly dependent on location services. The need to provide accurate indoor location services is becoming more and more urgent [1,2,3,4,5]. Indoor positioning technologies based on various types of sensors, such as Wi-Fi [6], Bluetooth [7], cameras [8], and inertial sensors [9], are rapidly developing. Since camera sensors can provide rich visual information on the environment and cameras can be obtained easily universally, vision-based indoor positioning technology [10,11] is increasingly receiving attention. According to the working modes of a camera, they can be divided into three categories: monocular, stereo, and RGB-D. The monocular camera has only one camera with the advantages of simple structure and low cost but has the disadvantage of scale uncertainty. The purpose of the stereo camera and the RGB-D camera is to overcome the shortcoming of the monocular mode—the inability to determine distance. The stereo camera consists of two cameras. We can use the distance between the two cameras to estimate the spatial position of each pixel, a process that is very similar to the human eye. The RGB-D camera can obtain the depth information by physical measurement, so it can save lots of calculations compared to the stereo camera. However, the RGB-D camera has many problems such as large noise, small field of view, easy exposure to sunlight, and the inability to measure transmission materials. Thus, we chose the stereo camera as the sensor for our vision-based indoor positioning system.

Vision-based indoor positioning technology can be divided into visual odometry (VO) [5] and visual-inertial odometry (VIO) [11]. The VO estimates the motion of a camera based on the movement of features in the captured image. The architecture of VIO includes two main components: the front-end and the back-end. The front-end abstracts sensor data into models that are amenable for estimation, while the back-end performs estimations of position on the abstracted data produced by the front-end. The VO can be classified into two types: the feature method and the direct method [12] according to whether features need to be extracted at the front-end. The front-end, based on the feature method, is a mainstream visual odometer, such as ORB-SLAM [13] and S-PTAM [14]. However, it is difficult to handle dynamic obstacles with VO, and it is very susceptible to environmental influences. An inertial measurement unit (IMU) can mitigate the effects of dynamic objects by receiving motion information. While an IMU can measure angular velocity and acceleration, there is a significant drift in these measurements which makes the pose obtained by the two times of integration of an IMU very unreliable. Camera data can effectively estimate and correct drift caused by an IMU. Although the use of cameras and inertial sensors are challenging because of dynamic obstacles in the line of sight and drift accumulation [12], respectively, the integration of these two sensors can compensate for their shortcomings and provide more accurate positioning solutions [15,16]. The VIO provides a reliable choice for indoor location services that are based on the complementarity between the IMU and the camera. A pedestrian positioning system with a wearable camera and an inertial sensor is proposed in Reference [17]. The relative displacement is estimated by dead reckoning based on inertial measurements, and the cumulative error is corrected by image matching. Li et al. [18] proposed a 3D motion tracking and reconstruction system on a mobile device using a camera and IMU. According to their pose estimation methods, VIO can be divided into two categories: the filter estimation method and the optimization-based estimation method. Filter-based classic VIOs include multi-state constraint Kalman filter (MSCKF) [19] and robust visual-inertial odometry (ROVIO) [20]. A VIO based on an optimization method is the visual-inertial state estimator (VINS) [21]. 

Vision-based positioning systems are very susceptible to environmental influences. The ability to extract stable and accurate visual observations is a key factor affecting positioning accuracy. When visual observations (image features) are rare or unevenly distributed, the positioning error of a vision-based positioning system can be very large and can even lead to system collapse. Alexander [22] proposed an efficient method of integrating IMU data to overcome these problems of visual observations. This work exploited IMU data to predict the movement which obtained the initial guess of the camera by assuming constant velocity. Tashfeen [23] proposed an extended Kalman filter (EKF)-based loosely coupled 3D inertial sensor system and a VO integration scheme. However, the estimation result of an EKF is dependent on the quality of visual observations. Some studies [24] have focused more on the system and algorithmic robustness rather than quantitative and verifiable integrity, particularly for feature-based processing. Veth [25] introduced the concept of regional bounding for feature correspondence among time-sequenced image frames, and he included some unique feature criteria that can provide some protection from feature correspondence errors. Koch [26] and Kyriakoulis [27] focused on using additional vision measurements such as colors. However, these methods only improve the quality of visual observations and do not solve the problem of rare visual observations.

Pedestrian dead reckoning (PDR) systems [28,29], which contain the data of accelerometer and gyroscope, can provide relative positioning information, velocity, and orientation in an indoor inertial navigation system. Jimenez [30] conducted extensive research on the pedestrian dead reckoning algorithm using IMU. Lee [31] proposed an experimental heuristic approach to multi-pose PDR indoor positioning for structured environments which reduce the heading error considering the effects of turning and hand-shaking events based on identifying six poses and four modes. However, PDR will generate cumulative errors resulting in unreliable positioning results under long-term operation. Yan [32] proposed a method that integrates VO and PDR which matches the time steps of positioning information from VO and PDR for inertial positioning calibration purposes. This fusion matching method still preserves the error of positioning caused by VO when the quality of visual observation is poor. Dae [33] introduced a selective integration method to improve positioning accuracy under GNSS (Global Navigation Satellite System)-challenged environments when applied to multiple navigation sensors. The weighted least squares method was applied to derive the performance index which only measures the goodness of geometrical distributions of feature points. The method does not consider the case in which feature points are sparse and moving.

In this paper, we analyzed the error source and divided it into four error situations when the vision-based positioning system had a large positioning error under special environments. We proposed autonomous integrity monitoring of a visual observation-based pedestrian dead reckoning system. According to the characteristic of short-term reliability of PDR [34], PDR can output positioning results when the visual observation is unreliable. Results from experiments show that our positioning system is more robust to indoor environments having fewer textures, dynamic obstacles or low lightings. The main contributions of this research are summarized below:

1. We analyzed the error source and divided it into four error situations when the vision-based positioning system had a large positioning error under special indoor environments that had fewer textures, dynamic obstacles or low lightings.
2. We proposed autonomous integrity monitoring of a visual observation-based pedestrian dead reckoning system. According to the characteristic of short-term reliability of PDR, the proposed PDR-assisted visual integrity monitoring system switches states between VIO (or VO) and PDR automatically to provide more accurate positions in an indoor environment.

## 2. Background

**Figure 1:**
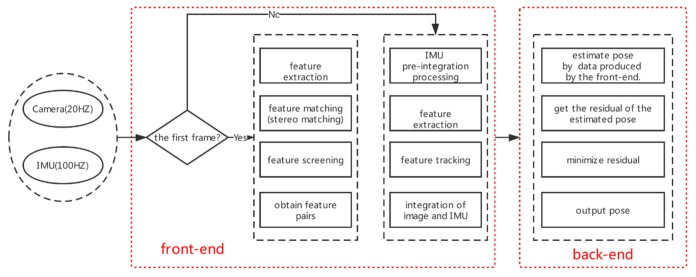
The full pipeline of the visual inertial odometer.

Visual inertial odometers generally consist of two parts, namely, front-end and back-end. The front-end mainly deals with a sensor’s observations such as feature extraction, feature tracking, feature screening, IMU pre-integration processing, and integration of images with IMU data. The back-end mainly performs estimations of position on the abstracted data produced by the front-end by minimizing the residual which is caused by observations through a filter or an optimization scheme. The structural block diagram of the system is shown in Figure 1.

The goal of the back-end is to estimate the 3D pose of the camera frame {*C*} for a global frame of reference {*G*}. Since a stereo camera consists of two cameras, the camera frames are represented by {$C_{k},k=1\;or\;2$}. To make it clearer and simpler to analyze the impact of visual observations on pose estimation, we defined the state vector and observation of the positioning system. The state vector $X_{k}$ at time-step k in visual-inertial odometer can be defined as Equation (1), including the evolving state $X_{IMU_{k}}$ of the IMU and the camera pose (attitude ${}_{\;G}^{C_{k}}\bar{q}$ and position ${}^{G}p_{C_{k}}$).

(1) $$X_{k}={\left[\begin{array}{ccc} X_{IMU_{k}}^{T} & {}_{\;G}^{C_{k}}\bar{q}{}^{T} & {}^{G}p_{C_{k}}^{T} \end{array}\right]}^{T}\begin{array}{cc} , & where\;X_{IMU}={\left[\begin{array}{ccc} {}_{\;\;G}^{IMU}\bar{q}{}^{T} & b_{g}^{T} & \begin{array}{ccc} {}_{\;}^{G}v{}_{\;}^{T} & b_{a}^{T} & {}_{\;}^{G}p{}_{\;}^{T} \end{array} \end{array}\right]}^{T} \end{array}$$

where

$${}_{\;\;G}^{IMU}\bar{q}{}^{T}$$

is the rotation from frame {*G*} to frame {*IMU*}, ${}_{\;}^{G}v$ and ${}_{\;}^{G}p$ are the IMU position and velocity with respect to {*G*}, $b_{g}$ and $b_{a}$ are the biases of the gyroscope and accelerometer measurements. 

The *k*-th measurement of the camera contains a series of feature points that are observed by the *k*-th camera pose (${}_{\;G}^{C_{k}}\bar{q},{}^{G}p{}_{C_{k}}^{\;}$). The measurement model of a feature point $z_{i}$ is expressed by the following equation:

(2) $$z_{i}=\frac{1}{{}^{C}Z_{i}}\left[\begin{array}{c} {}^{C}X_{i}\\ {}^{C}Y_{i} \end{array}\right]+n_{i},\begin{array}{cc} & i\in f,C \end{array}\in \{C_{1},C_{2}\}$$

where f represents a collection of all feature points, $C_{1}\;\mathrm{and}\;C_{2}$ represent the left and right cameras, respectively, and $n_{i}$ is the 2 × 1 image noise vector. The feature position expressed in the camera frame, ${}^{C}P{}_{i}$, is given by:

(3) $${}^{C}p{}_{i}=\left[\begin{array}{c} \begin{array}{c} {}^{C}X_{i}\\ {}^{C}Y_{i} \end{array}\\ {}^{C}Z_{i} \end{array}\right]=C\left({}_{G}^{C_{i}}\bar{q}\right)\left({}^{G}p{}_{i}-{}^{G}p{}_{C}\right)$$

where ${}^{G}P{}_{i}$ is the 3D feature position in the global frame, ${}^{G}P{}_{c}$, is the camera in the global frame and $C\left({}_{\;G}^{C_{i}}\bar{q}\right)$ is the rotation matrix between the camera frame and the global frame. Once the estimate of the feature position is obtained, we can compute the measurement residual:

(4) $$r_{i}=z_{i}-{\hat{z}}_{i}≃H_{C}\tilde{X}+H_{i}{}^{G}\tilde{p}{}_{i}+n_{i},\begin{array}{c} where \end{array}\left\{ \begin{array}{l} H_{C}=\frac{\partial z_{i}}{\partial {}^{C_{1}}p{}_{i}}\cdot \frac{\partial {}^{C_{1}}p{}_{i}}{\partial X_{C_{1}}}+\frac{\partial z_{i}}{\partial {}^{C_{2}}p{}_{i}}\cdot \frac{\partial {}^{C_{2}}p{}_{i}}{\partial X_{C_{1}}}\\ H_{i}=\frac{\partial z_{i}}{\partial {}^{C_{1}}p{}_{i}}\cdot \frac{\partial^{C_{1}}p{}_{i}}{\partial {}^{G}p{}_{i}}+\frac{\partial z_{i}}{\partial {}^{C_{2}}p{}_{i}}\cdot \frac{\partial {}^{C_{2}}p{}_{i}}{\partial {}^{G}p{}_{i}} \end{array}\right.$$

where $H_{c}$ and $H_{i}$ are the Jacobians of the measurement $z_{i}$ for the state and the position estimate of the feature. With all the sets of measurement equations formed by the feature points, we can obtain the optimal solution by minimizing the error and get the optimal position estimate.

(5) $$\min{\|r-H[\begin{array}{c} \tilde{X}\\ {}^{G}\tilde{p}{}_{i} \end{array}]\|}^{2}\begin{array}{cc} , & H \end{array}=\left[\begin{array}{c} \begin{array}{c} \begin{array}{c} \begin{array}{cc} H_{C} & H_{1} \end{array}\\ \begin{array}{cc} H_{C} & H_{2} \end{array} \end{array}\\ ... \end{array}\\ \begin{array}{cc} H_{C} & H_{i} \end{array} \end{array}\right]$$

## 3. Visual Error Analysis and Autonomous Integrity Monitoring

### 3.1. Visual Error Analysis

Large errors have been observed in the positioning results under special environments such as fewer textures, low lightings or dynamic obstacles [35]. In this research, we closely investigated the error source occurring in four scenarios: an indoor environment with fewer textures resulting in insufficient features; an indoor environment with dim lighting causing the failure of feature tracking; an indoor environment with uneven textures resulting in an uneven distribution of features; and am indoor environment with dynamic obstacles producing moving features.

#### 3.1.1. Insufficient Features

Commonly used feature extraction algorithms include the Scale-Invariant Feature Transform(SIFT) [11], Speed Up robust Feature Transform (SUFT) [12], Features from Accelerated Segment Test (FAST) [13] and Oriented feature from accelerated segment test and Rotated Binary robust independent elementary features (ORB) [14] algorithms. These algorithms are often used in processing VIO projects. In an image, a point with a strong contrast of surrounding pixels is defined as a feature point. The contrast of point P can be expressed as:

(6) $$V\left(x,y\right)=\sum \left|I\left(x+\Delta x,y+\Delta y\right)-I\left(x,y\right)\right|\begin{array}{cc} , & V\left(x,y\right)∼\sum \left[I_{x}{}^{2}\cdot \Delta x^{2}+I_{y}{}^{2}\cdot \Delta y^{2}+2I_{x}I_{y}\cdot \Delta x\Delta y\right] \end{array}$$

where x and y represent the pixel coordinates of P. I(x,y) and V(x,y) represent the gray value and contrast of the point, respectively. The value of *V* mainly depends on the gradient of the point P in the *x* and *y* directions $\left(I_{x}\;\mathrm{and}\;I_{y}\right)$. The larger the gradient value, the easier it is to be detected by the detector. It is difficult to obtain sufficient feature points from scenes having fewer textures (i.e., white walls) or dim lightings which is common in indoor environments. Position estimation can be performed when the feature point pairs exceed eight pairs [36].

(7) $$\left[\begin{array}{c} \delta \hat{X}\\ \delta {}^{G}\hat{p}{}_{i} \end{array}\right]=(H^{T}H{)}^{-1}H^{T}r$$

When the number of feature points is sufficient, rank(H)≥8, the constraint Equation (7) is sufficient to obtain the optimal solution. When the number of feature points is insufficient, the constraint condition is insufficient, and the estimated value error $\left(\delta \hat{X}\;\mathrm{and}\;\delta {\hat{p}}_{i}\right)$ becomes larger. This leads to an increase in the positioning error:

(8) $$\hat{X}=X+\delta \hat{X}\begin{array}{cc} , & {}^{G}\hat{p}{}_{i}={}^{G}p{}_{i}+\delta {}^{G}\hat{p}{}_{i} \end{array}$$

#### 3.1.2. Lighting Causes the Failure of Feature Tracking

Illumination changes often occur in indoor environments, and we used the Lambertian model as the lighting model.

(9) $$I\left(x,y\right)=\rho \left(x,y\right)\cdot h{\left(x,y\right)}^{T}\cdot S$$

where I(x,y) is the image gray value, ρ(x,y) is the object reflectivity, h(x,y) is the surface normal vector, and S is the lighting intensity. We found that with feature tracking, it is easy to lose leads to inaccurate positioning during lighting changes. The optical flow method is based on the assumption that the gray level is unchanged. Substitute the lighting formula:

(10) $$\frac{\partial I}{\partial x}\frac{dx}{dt}+\frac{\partial I}{\partial y}\frac{dy}{dt}=-\frac{\partial I}{\partial t}\to \left[\begin{array}{cc} I_{x} & I_{y} \end{array}\right]\left[\begin{array}{c} \mu \\ \nu \end{array}\right]=-\rho \cdot \left[\frac{\partial h^{T}}{\partial t}\cdot S+\frac{\partial S}{\partial t}\cdot h^{T}\right]$$

where $I_{x}$ and $I_{y}$ are the gradient values of the feature points in the x and y directions, respectively, and μ and v are the velocity of the motion in the x and y directions, representing the feature points. As shown in Equation (11), the residual $\delta r_{i}$ of the features will become larger, while the light intensity changes and ∂S/∂t become larger.

(11) $$\delta r_{i}=\frac{1}{{}^{C}Z{}_{i}}\left[\begin{array}{c} {}^{C}X{}_{i}-({}^{C}X{}_{i}+\mu t)\\ {}^{C}Y{}_{i}-({}^{C}Y{}_{i}+\nu t) \end{array}\right]=\frac{t}{{}^{C}Z{}_{i}}\left[\begin{array}{c} I_{x}\\ I_{y} \end{array}\right]\cdot \left[\rho \frac{\partial S}{\partial t}\cdot h^{T}\right]$$

#### 3.1.3. Uneven Distribution of Features

**Figure 2:**
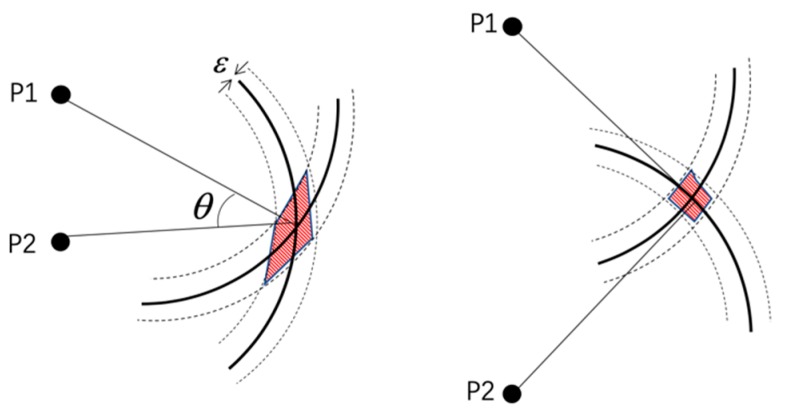
The error diagram of the geometric relationship of the feature points. The accuracy of the position estimation depends on the error in the pose estimation and geometric angle of the observation, and the shaded part indicates the uncertainty of the position estimation.

It can be seen in the observation equation of the image that the presence of noise causes positional errors in the feature points in the image. The position error of the feature points will affect the state estimation of the camera when calculating the re-projection error. To better represent the role of the geometric relationship between image feature points and camera poses, our line used a simple two-dimensional example to describe the geometric relationship. As shown in Figure 2, P1 and P2 represent two picture feature points. If there is no noise influence, the camera pose can determine the position by the intersection of two circles with two feature points as the center and two projection distances. But the measurement was not ideal, and the uncertainty of the noise was ±ε.

We describe the quality of the position estimate based on the camera state’s Jacobian matrix of feature points $H_{i}$. Assume that the measurement error is zero-average, the positioning error is also zero-average. Then we can obtain the expected value E(ΔX) and covariance Cov[ΔX] of the error in the position calculation.

(12) $$E\left(\Delta X\right)=E\left(\hat{X}-X\right)=0\begin{array}{cc} , & Cov\left[\Delta X\right]=\sigma^{2}{\left(H_{i}{}^{T}H_{i}\right)}^{-1}\sqrt{b^{2}-4ac} \end{array}$$

The amount of change in the position error in the x, y, and *z* directions is represented by $\sigma_{x}^{2},\;\sigma_{y}^{2},\;\sigma_{z}^{2}$, respectively. $H_{ii}$ is used to represent the first element on the diagonal in the diagonal matrix ${\left(H_{i}^{T}H_{i}\right)}^{-1}$. Then, it can be expressed as:

(13) $$SD(\Delta X)=\sqrt{\sigma_{x}{}^{2}+\sigma_{y}{}^{2}+\sigma_{z}{}^{2}}=\sqrt{H_{11}+H_{22}+H_{33}}$$

#### 3.1.4. Moving Features

All moving objects, such as pedestrians or vehicles, will affect the positioning result during positioning. When the feature points are concentrated on the moving object, the relative movement of the feature points results in a larger calculated camera movement. This situation can be expressed in the world coordinates of the feature points as having an additional motion shift, $\Delta {}^{G}P{}_{f_{j}}$, which affects the camera’s observation as shown in Equation (14):

(14) $$\left[\begin{array}{c} \begin{array}{c} {}^{C_{i}}X{}_{j}+\Delta x\\ {}^{C_{i}}Y{}_{j}+\Delta y \end{array}\\ {}^{C_{i}}Z{}_{j}+\Delta z \end{array}\right]=C\left({}_{\;G}^{C_{i}}\bar{q}\right)\left({}^{G}p{}_{f_{j}}+\Delta {}^{G}p{}_{f_{j}}-{}^{G}p{}_{C_{i}}\right)$$

Let us analyze the residuals $r_{i}$ generated by the offset (Δx,Δy,Δz) of the feature.

(15) $$r_{i}=\frac{\Delta z}{{}^{C}Z{}_{i}({}^{C}Z{}_{i}+\Delta z)}\left[\begin{array}{c} {}^{C}X{}_{i}\\ {}^{C}Y{}_{i} \end{array}\right]-\frac{1}{{}^{C}Z{}_{i}+\Delta z}\left[\begin{array}{l} \Delta x\\ \Delta y \end{array}\right]$$

### 3.2. PDR-Assisted Visual Integrity Monitoring

Although PDR has a problem of cumulative error, the error over a short time is very small. The rotation matrix of the IMU relative to the world coordinate system can be constructed by the three-axis gyroscope. After the three-axis acceleration is rotated, the relative position information (x,y,z) can be obtained by performing the integral operation as shown in Equation (16).

(16) $$s\left(t+\Delta t\right)=s\left(t\right)+v\left(t\right)\Delta t+\frac{1}{2}a^{\prime }\Delta t^{2}$$

Now assume that there are two sampling points $O_{1},\;O_{2}$, the sampling interval is Δt and the velocity of time $O_{1}$ is ν, the displacement is s, and the state covariance matrix is $P_{1}=\left[\begin{array}{c} P_{11}\;\;\;\;P_{12}\\ P_{21}\;\;\;\;P_{22} \end{array}\right]$. Accelerometer observations can cause inaccurate deviations due to the shocks generated during motion. We first analyzed the one-dimensional motion, the acceleration at time $O_{1}$ is $f_{mea}=f_{true}+\delta f$. An estimated value of the state quantity at $O_{2}$ can be obtained from the state quantity at $O_{1}$. The deviation caused by δf is:

(17) $$\left[\begin{array}{c} \delta s\\ \delta v \end{array}\right]=\left[\begin{array}{c} \delta f\cdot \Delta t\left(\frac{1}{2}\Delta t-\frac{p_{12}+\Delta t\cdot p_{22}}{p_{22}+R}\right)\\ \delta f\cdot \Delta t\left(1-\frac{p_{22}}{p_{22}+R}\right) \end{array}\right]$$

where R is the covariance matrix of the observed noise.

**Figure 3:**
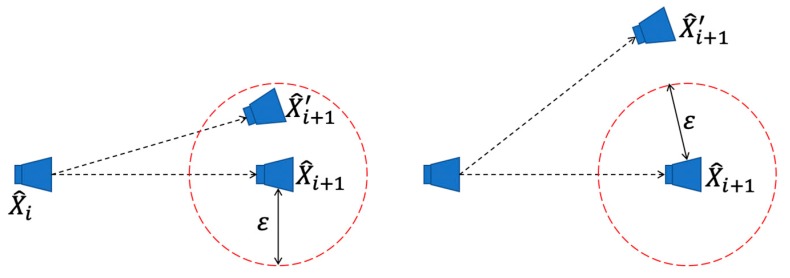
Pedestrian Dead Reckon-assisted visual integrity testing. ε is the error range of PDR positioning. When the positioning result of the VIO system exceeds the error range of the PDR, the PDR result is used instead of the VIO result.

In this paper, we propose an autonomous PDR-assisted visual integrity monitoring approach to improve positioning accuracy. According to the characteristic of short-term reliability of PDR, the proposed PDR-assisted visual integrity monitoring system switches states between VIO (or VO) and PDR automatically to provide a more accurate position in an indoor environment. The specific switching situation is shown in Figure 3. When the positioning result of the VIO system exceeds the error range of the PDR, the PDR result is used instead of the VIO result. ${\hat{X}}_{i}$ is the camera pose at the ith moment, ${\hat{X}}_{i+1}$ is the camera pose at the i+1th moment obtained by PDR, and ${\hat{X}}_{i+1}^{\prime }$ is obtained by VIO. ε is the error range of PDR. 

Hypothesis deviation obeys Gaussian distribution e~N(0,∑). Now, e is a three-dimensional vector. In order to facilitate the calculation, the inner product of the computation vector is transformed into a scalar.

(18) $$r=e^{T}\sum e={\left(\sum^{-\frac{1}{2}}e\right)}^{T}\left(\sum^{-\frac{1}{2}}e\right),where\left(\sum^{-\frac{1}{2}}e\right)∼N\left(0,I\right)$$

It is a normal distribution of multidimensional standards. It can be thought of as the sum of the squares of two independent random variables subject to the standard normal distribution which obeys the chi-square distribution of three degrees of freedom. The probability distribution (cumulative distribution function) is a=F(x), and given an a, we can determine an interval $\left[0,F^{-1}\left(a\right)\right]$. $F^{-1}\left(a\right)$ is the threshold we are looking for to determine the visual integrity. The above is the theoretical analysis part of the threshold of a PDR-assisted visual integrity monitoring approach. 

Our indoor positioning system is based on the Multi-State constrained Kalman Filter (MSCKF) positioning algorithm and PDR. It is very important to switch states between MSCKF and PDR automatically to provide an accurate positioning result for our indoor positioning system. The problem that visual observations cause on MSCKF can be attributed to the fact that visual observations are not updated or are updated incorrectly. So, the update frequency of visual observations and the estimation of the gyroscope bias will generate a large abnormal fluctuation when the abnormality is detected by PDR-assisted visual integrity monitoring (as shown in Figure 9). We used the update frequency of visual observation $f_{update}$ and the estimation of the gyroscope bias ${\hat{b}}_{gyr}$ to switch states between MSCKF and PDR automatically as shown in Equation (19):

(19) $$P_{out}=\{\begin{array}{l} P_{PDR}\;\;\;\;\;\;\;\;\;\;{\hat{b}}_{gyr}>\tau_{1}\;\&\&\;f_{update}<\tau_{2}\\ P_{MSCKF}\;\;\;\;\;\;\;\;\;\;\;\;\;\;\;\;\;\;\;\;others\;\;\;\;\;\;\;\;\;\;\;\;\;\;\;\;\;\;\;\;\;\; \end{array}$$

where $P_{out}$ is the estimated poses of our positioning system, $P_{PDR}$ and $P_{MSCKF}$ represent the estimated poses of PDR and MSCKF, respectively. $\tau_{1}$ and $\tau_{2}$ are the hyperparameters of the system to switch states between MSCKF and PDR automatically. MSCKF needs to re-measure the observations to prevent the impact of visual observation errors on later operations at each switch. During the operation of the system, the MSCKF cannot be restored to normal immediately after replacing the attitude angle. To prevent the wrong gyroscope bias from affecting the subsequent pose estimation, we will continue to output the estimated poses of PDR for some time. That time period will be used to allow MSCKF to re-measure the observations and complete the restart operation. 

## 4. Experiments and Evaluation

**Figure 4:**
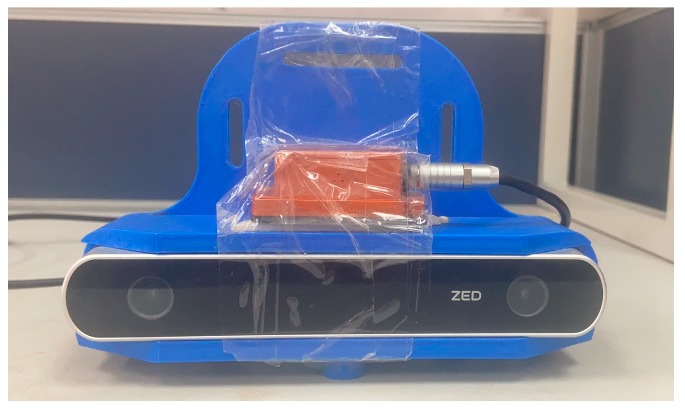
The device used for the indoor experiment. It contains one stereo camera (ZED, 30 HZ) with a 672 × 376 resolution.

The sensor suite we used is shown in Figure 4. It consists of a stereo camera (ZED, 30 HZ from stereolabs, San Francisco, U.S.A) and an IMU (MTi-100 from XSENS, Netherlands). The indoor positioning system was based on the MSCKF positioning algorithm and PDR. In data collection, the sensor suite was held in hands, and the participant walked in an indoor environment. The first part of the experiment assessed the scenarios’ impact on the positioning results. The second part of the experiment tested and evaluated the proposed PDR-assisted visual integrity monitoring which switches states between MSCKF and PDR automatically to provide an accurate position.

### 4.1. Assessing Environment Impacts

The following experiments were designed to evaluate the above four causes of errors identified in the previous session.

#### 4.1.1. Insufficient Features

**Figure 5:**
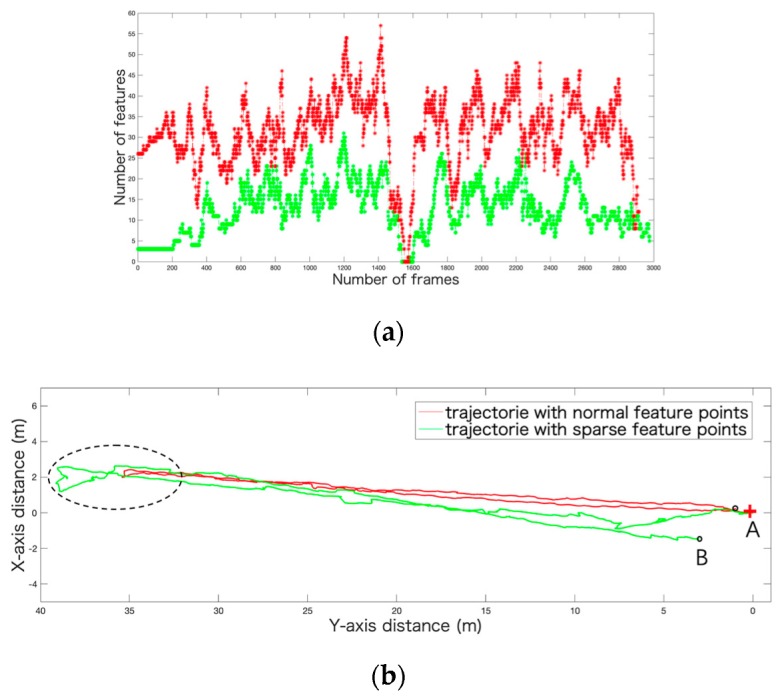
(**a**) The red line is the number of feature points with the threshold set to 20 per frame, and the green line is the number of sparse features with the threshold set to 60 per frame. (**b**) The red cross marks the starting point, point A is the endpoint of the track in the original state of the feature point, and point B is the endpoint of the track when feature points were sparse.

As shown in Figure 5a, we changed the threshold for the number of feature points per frame for the same set of data to extract feature points. Figure 5a showed that the lower the threshold, the more the number of feature points. For both thresholds of 20 and 60, the number of feature points was 0 in the 1560th frame. This is because a white wall was encountered, and the feature points could not be extracted. We drew the corresponding positioning trajectory as shown in Figure 5b. When the feature points were scarce, the camera’s ability to correct the IMU was weaker, the path was not serrated enough, and the trajectory also showed significant deviations between the *x*-axis and the *y*-axis.

#### 4.1.2. Lighting Causes the Failure of Feature Tracking

**Figure 6:**
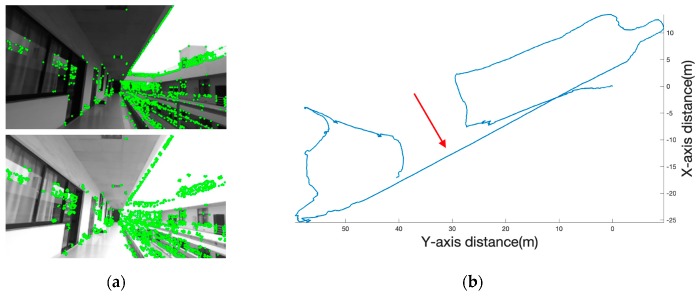
(**a**) The average gray value of the top image is 97.0845, the average gray value of the bottom image is 183.946, and the number of features extracted by the top image is 946. The number of features extracted by the bottom image is 1543. (**b**) A long line pointed by the red arrow is that the different light of the left and right cameras resulted in no feature points and serious deviations in the trajectory.

When the lighting is different from the left and the right cameras, the average gray value of the images acquired by the left and right cameras was different, and the matching rate was low. Figure 6a is a feature point distribution map obtained by FAST feature extraction on the images acquired by the left and right cameras. However, the image matching rate of the left and right cameras was not high, and the matching ratio was only 0.55. No feature points exist in the image after stereo matching. If the feature detection module does not have the feature point data output, the visual inertia mileage calculation method cannot perform the posture update, resulting in the track accumulating offset, and serious errors may occur as shown in Figure 6b.

#### 4.1.3. Uneven Distribution of Features

**Figure 7:**
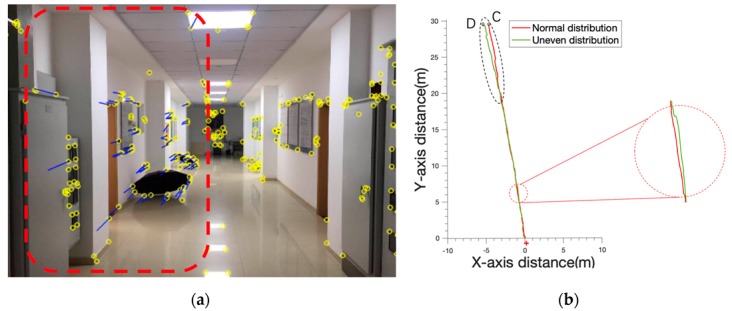
(**a**) The feature point distribution is controlled in the red area. The yellow circle represents all the extracted feature points, and the blue line segment represents the tracking track of the feature points. (**b**) The red cross marks the starting point, the C point is the endpoint of the track where the feature point distribution was normal, and the D point is the track end where the feature point was unevenly distributed.

We used the distribution of feature points as variables and compared the trajectory with the original feature distribution as shown in Figure 7a. However, when the feature points were only distributed in the red area, the movement trajectory of the feature points was directed to the right side of the image. As shown in Figure 7b, the trajectory of the feature with uneven distribution had an obvious deviation to the left.

#### 4.1.4. Moving Feature Point

**Figure 8:**
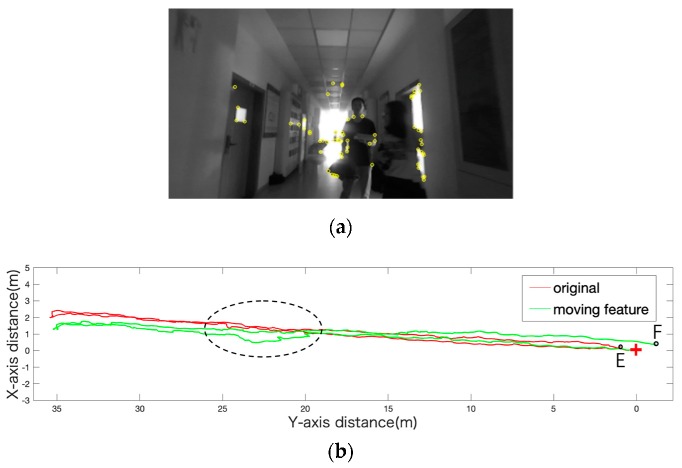
(**a**) Open circles represent moving feature points; solid circles represent stationary feature points. (**b**) The red cross marks the starting point and the E point is the original track. The point F is the endpoint of the trajectory affected by the moving feature points.

Pedestrians walked in front of the camera, and the contrast tracking is plotted in Figure 8b. It is obvious in the circle that the green track shifted to the left because of the influence of the pedestrians. We analyzed the details of this moment. As can be seen from Figure 8a, when the pedestrian moved, more than half of the extracted feature points were gathered on the pedestrian. Therefore, the movement of the pedestrian relative to the camera will lead to the deviation of the positioning results. As the pedestrian moved toward the right side of the camera, those feature points on the pedestrian accumulated the corresponding movements which caused the estimated position to produce a leftward deviation as shown in the black elliptical region in Figure 8b.

### 4.2. Evaluation of Proposed PDR-Assisted Visual Integrity Monitoring

**Figure 9:**
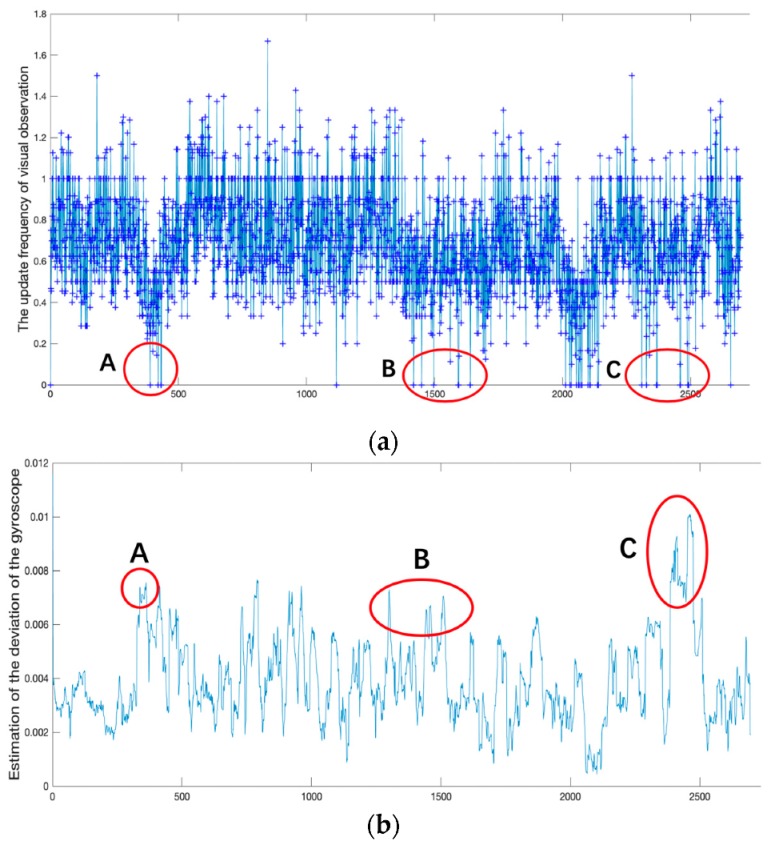
(**a**) The curve of the update frequency of visual observations; (**b**) the estimation of the gyroscope bias.

This experiment was carried out in a large office building with a length of 100 m and a height of 20 m which lasted for 20 min and spanned three floors. The abnormalities of visual observations led to the update frequency of visual observation, and the estimation of the gyroscope bias generated a large abnormal fluctuation. According to the update frequency of visual observation and the estimation of the gyroscope bias, we divided the path into three parts to introduce the effect of PDR-assistance based on visual observations as shown in Figure 9 (Section A, B, C). The following are the content tests and evaluations of the experimental results of our positioning system with the proposed PDR-assisted visual integrity monitoring.

#### 4.2.1. Section A

**Figure 10:**
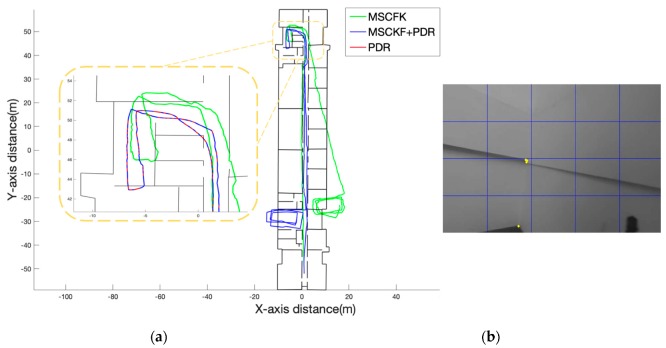
(**a**) Comparison of the trajectories of the Multi-States Constrained Kalman Filter (MSCKF) and MSCKF+PDR. The green color is the MSCKF and the blue color is the MSCKF+PDR. The yellow area is an enlarged view of the path of the stair area, where the red part is the output path of the PDR assisted. (**b**) The picture of the stair scene and the yellow point is the extracted feature point.

The length of walking distance of “Section A” was approximately 340 m. The specific action track was to walk straight in the corridor, then go downstairs to the next floor and walk three times in the hall. It can be seen from Figure 10a that the path of the MSCKF had a large deviation in direction. To illustrate the change in the trajectory, the path in the yellow area was the amplified path, and the red path was the path of the PDR assistance. The scene here is shown in Figure 10b, which is the stair area. When the feature observation is rare and a turn is made, the visual update frequency of the MSCFK will become lower, which means the frequency of the feature points discarded is not enough during the filter update process. These situations cause a deviation in the direction of the MSCKF. After the auxiliary switching by PDR, it can effectively improve the system to provide a reliable path output when a visual observation is insufficient.

#### 4.2.2. Section B

**Figure 11:**
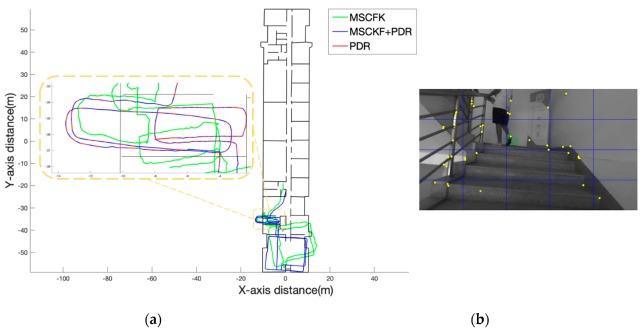
(**a**) Comparison of the trajectories of MSCKF and MSCKF+PDR. (**b**) The pedestrian walking, the yellow points are the extracted feature points, and the green is the tracking path of the feature point.

The length of walking distance of “Section B” was approximately 214 m which went upstairs to the rooftop area and involved two laps of the rooftop space. As shown in Figure 11a, the path of the MSCKF was a little irregular which is the yellow area. We can see in Figure 11b that moving feature points always exist in the process of going upstairs. There were many failures of feature tracking on the stairs, resulting in the MSCKF’s trajectory direction always being offset. In this case, the relative displacement based on the prediction of IMU and visual observation was quite different. According to this situation, the frequency of the switching of the PDR was relatively high, and the positioning direction could be ensured more accurately. But there was an error in the calculation of the step size of the PDR, resulting in a longer overall trajectory in the process of going upstairs.

#### 4.2.3. Section C

**Figure 12:**
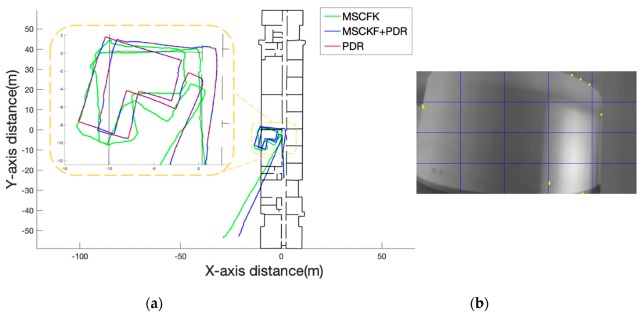
(**a**) Comparison of the trajectories of MSCKF and MSCKF+PDR. (**b**) The picture of the stair scene and the yellow points are the extracted feature points.

The length of walking distance of “Section C” was approximately 166 m, and the route was looped two times in an empty room which mostly contained turns. Due to the existence of a large number of white wall scenes, the visual observation of the MSCKF was relatively poor in quantity and quality. The filter estimated the deviation of a wrong gyroscope, resulting in a deviation of the overall trajectory direction. With the short-term reliability of the PDR, the direction deviation of the positioning can be reduced. It can be seen from Figure 12a, that, at the first turn, there was no PDR assistance, resulting in a deviation in the direction of the MSCKF. However, it was obvious that the PDR switched at the turn the second time which effectively reduced the direction error of the system.

**Figure 13:**
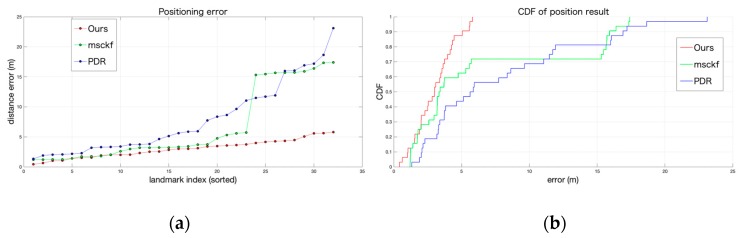
(**a**) Comparison of the positioning error of MSCKF, PDR, and our system. (**b**) CDF of the position result of MSCK, PDR, and our system.

To describe the positioning results more clearly, we labeled 32 landmarks and recorded the location information of those landmarks. Then we calculated the positioning errors of MSCKF, PDR, and our positioning system based on the experimental data. As shown in Figure 13, it can be seen from the line chart of the positioning error and the graph of the cumulative distribution function (CDF) that the positioning accuracy of our system was significantly improved. The positioning error of the MSCKF was mainly caused by the poor quality of visual observation, while the positioning result of PDR was due to the cumulative error that was caused by the error of step detection during pedestrian turning.

**Figure 14:**
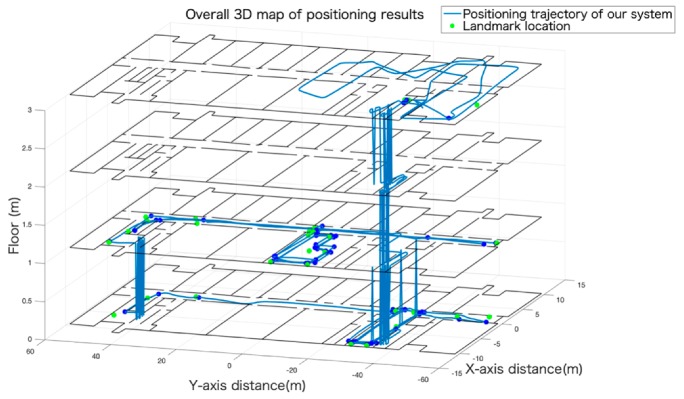
The map had four floors. Green dots represent real landmark points that were calibrated in advance. The blue route is the positioning result of our system.

At this part, we performed a real-time positioning experiment, such as IPIN [37] (The International Conference on Indoor Positioning and Indoor Navigation) competitions, and conducted a quantitative analysis of the positioning situation based on the criteria for performance evaluation of IPIN competitions. As shown in Figure 14, we tested our system in a large and challenging multi-floor environment with a significant path length and duration to evaluate its performance. The total length of the walking route was 1400 m, and the walking area spanned four floors. Then, we performed a numerical analysis to show the accuracy of our system in detail.

**Figure 15:**
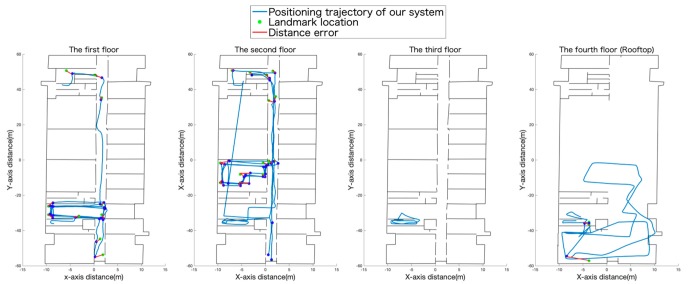
The positioning results of each floor are displayed. The blue track is the positioning result of our system, and the green point is the real landmark point. The red line indicates the error distance between the actual landmark and the system anchor point.

**Figure 16:**
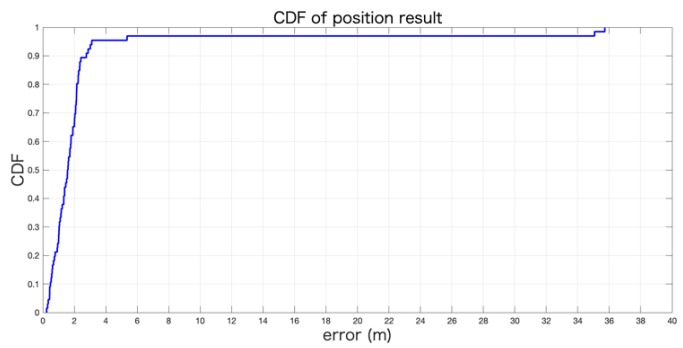
The CDF of the position result of our system.

To better display the experimental results, the positioning trajectory of each floor is shown in Figure 15. The average error of the positioning results in this experiment was approximately 2.5838 m; we also plotted the CDF of the positioning error as shown in Figure 16. The final score metric was the third quartile of the positioning error in IPIN which makes the accuracy results less prone to the influence of outliers and more in-line with demanded accuracy for commercial systems. So, the final score of our system was 2.13 m. 

## 5. Summary and Discussion

To solve the problem of vision-based positioning systems being very susceptible to environmental influences, we analyzed the error source of visual observations when vision-based positioning systems had a large positioning error under special indoor environments having fewer textures, dynamic obstacles or dim lighting. We divided the error sources of visual measurement into four error situations and performed detailed analysis and explanation. The first part of the experiment assessed the scenarios’ impact on the positioning results to display intuitively the effect of feature observation. To address this issue, we proposed autonomous integrity monitoring of visual observation based on a pedestrian dead reckoning system. Through the error analysis of PDR, it was found that the error of PDR in a short time was small and bounded. According to the characteristic of short-term reliability of PDR, the proposed PDR-assisted visual integrity monitoring switches states between MSCKF and PDR automatically to provide a more accurate position in an indoor environment. The second part of the experiment tested and evaluated the proposed PDR-assisted visual integrity monitoring. In conclusion, we proved that our positioning system can effectively provide more reliable and accurate positioning results. Future research should consider the potential effects of visual observation more carefully. Also, future investigations are necessary to improve the accuracy of the step size of the PDR to improve the positioning accuracy of the system.
